# A New extractive spectrophotometric method for determination of rizatriptan dosage forms using bromocresol green

**DOI:** 10.1186/2008-2231-21-12

**Published:** 2013-02-02

**Authors:** Effat Souri, Abbas Kaboodari, Noushin Adib, Massoud Amanlou

**Affiliations:** 1 Department of Medicinal Chemistry, Faculty of Pharmacy and Drug Design and Development Research Center, Tehran University of Medical Sciences, Tehran, 14155-6451, Iran; 2 Department of Pharmaceutics, Food and Drug Laboratory Research Center, Ministry of Health, Tehran, Iran

**Keywords:** Rizatriptan, Bromocresol green, Lon-pair complexation, Spectrophotometry

## Abstract

**Background and the purpose of the study:**

Rizatriptan is used effectively for the treatment of migraine headache. In this study, a simple, rapid and low cost spectrophotometric method based on the ion-pair complexation is proposed for the determination of rizatriptan in raw material and dosage forms.

**Methods:**

The ion-pair complexation using bromocresol green as reagent was performed in a buffer solution and the absorbance was measured by a spectrophotometer. The ion-pair formation conditions were optimized and the accuracy and precision of the method were calculated.

**Results and major conclusion:**

Best results were achieved by using 6 ml of the bromocresol green reagent in the presence of phosphate buffer (pH 3.0). The stoichiometry of the resulted complex was 1:1. The within-day and between-day precision values were lower than 2.9 and 1.8 percent for the calibration range of 0.5–50 and 10–100 μg/ml, respectively. The proposed method was successfully used for the determination of rizatriptan in dosage forms without any interference.

## Introduction

**Figure 1:**
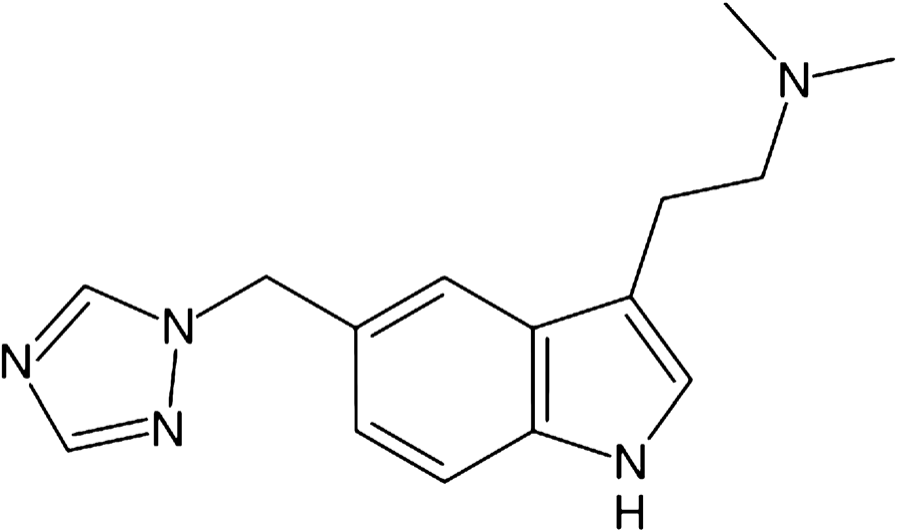
Chemical structure of rizatriptan.

Rizatriptan benzoate (Figure 1), N, N-dimethyl-5-(1H, 1, 2, 4-triazol-1-ylmethyl)-1H-indole-3-ethanamine benzoate, binds selectively with high affinity to human 5-hydroxytryptamine 1B/1D receptor. Clinical trials have demonstrated the effectiveness of oral rizatriptan for the treatment of migraine headache [1-3].

Several analytical methods based on liquid chromatography/ tandem mass spectrometry [4,5] or high performance liquid chromatography with fluorescence detection [6] has been reported for determination of rizatriptan in biological fluids. HPLC [7-9] and HPTLC [10] methods have also been used for quantitative determination of rizatriptan in bulk drug or pharmaceutical dosage forms. Furtheremore, few spectrophotometric methods were reported in the literature for the determination of rizatriptan in dosage forms [11-16]. Some of these methods are based on ion-pair complexation, redox-complexation reaction in the presence of iron ion and also oxidative coupling reactions. In most previously reported spectrophotometric methods a relatively narrow linear range or high limit of quantification has been obtained.

As rizatriptan is not official in USP or BP, there is still a demand for new simple, sensitive and rapid methods in order to determine the drug in pharmaceutical dosage forms. The present study deals with the development and validation of a sensitive extractive spectrophotometric method using bromocresol green (BCG) as ion-pair complexation reagent. The BCG reagent is relatively cheap and has been used before in our laboratory for determination of some other drugs [17-19]. Spectrophotometric methods are rapid, cost effective, simple and acceptable which use very routine and available instruments in every quality control laboratory. The validated method could be successfully used for routine quality control analysis without any special sample preparation before determination.

## Materials and methods

### Apparatus

A double beam UV-visible spectrophotometer (UV-160, Shimadzu, Japan) in the wavelength range of 200–800 was used for spectrophotometric measurements.

A Waters HPLC system consisting of a Model 515 pump, a Model 717 plus Autosampler and a Model 486 UV-visible detector (All from Waters, Milford, USA) were used. Multi-channel Chrom&Spec software for chromatography (version 1.5×) was used for data processing.

### Chemicals

Rizatriptan benzoate (purity 99.8%) was from Merck (Batch No: 000705126 0048038) and was kindly provided by Food and Drug Laboratory Research Center, Ministry of Health, Tehran, Iran. Bromocresol green (BCG) and the other analytical grade chemicals were purchased from Merck (Darmstadt, Germany).

### Standard solutions

A standard solution of rizatriptan benzoate (5 × 10^-4^ M) was prepared by dissolving 13.5 mg of the drug in 100 ml of distilled water.

A 5 × 10^-4^ M solution of BCG was prepared by dissolving 349 mg of BCG in 1000 ml of distilled water by adding 2 ml of 0.1 M NaOH for better solubility.

The phosphate buffer (0.1 M) was prepared by dissolving 1.78 g of NaH_2_PO_4_ in 1000 ml of distilled water. The pH value was adjusted to 3.0.

The Britton- Robinson buffers in the pH range of 2–7 were prepared from an equal mixture of 0.1 M acetic acid, 0.1 M boric acid and 0.1 M phosphoric acid. The pH value was adjusted using a 1 M NaOH solution.

### General procedure for sample preparation

An aliquot of 2 ml of rizatriptan solution (5 × 10^-4^ M), 1 ml of buffer solution (pH 3), 6 ml of BCG reagent (5 × 10^-4^ M), and 1 ml of distilled water were transferred to a 100 ml separating funnel. The mixture was extracted three times using 5, 3 and 2 ml portions of chloroform. The separating funnel was vigorously shacked for 30 seconds for extraction. The organic phase was separated and dehydrated by passing over anhydrous sodium sulfate and was transferred to a 10 ml volumetric flask. The volume was completed with chloroform and the absorbance was measured at 416 nm against a reagent blank.

### Optimization of the reaction conditions

#### Selection of the optimum pH

The effect of pH on ion-pair complex formation was studied by using the general procedure and Britton-Robinson buffers in the range of 2–7. Also, different kinds of buffers (phthalate, acetate, phosphate) at the same pH value were studied to find out the effect of buffer type.

#### Selection of the reagent amount

Using 2 ml of rizatriptan solution (5 × 10^-4^ M) and 1 ml of phosphate buffer (pH 3.0), different amounts of BCG reagent (0.5–7 ml) were added and after extraction, the absorbance of the resulting solution was measured at 416 nm.

#### Selection of the extracting solvent

To find out the best extracting solvent, chloroform; dichloromethane; ethyl acetate; and diethyl ether were used as extracting solvents and the absorbance of the resulting ion-pair complexes were compared.

#### The effect of the reaction time

The effect of time on the ion-pair complex formation was studied at various time intervals in the range of 0–60 min.

### Stoichiometric relationship

The Job’s method of continuous variations was applied to find out the stoichiometric ratio of the ion-pair complexation. Different volumes of rizatriptan and BCG, at equal molar concentrations (4 × 10^-4^ M), were mixed with a fixed total volume and the absorbance of the resulting ion-pair complex was measured according to the general procedure. The resulting absorbance was plotted over the mole ratio of rizatriptan.

### Determination of rizatriptan in dosage forms

Twenty Rizatriptan tablets (5 mg) (Farabi Pharmaceutical Co, Iran) were finely powdered using a mortar and pestle. Next, an amount of the resulted power equivalent to one tablet (5 mg of rizatriptan) was accurately weighed and transferred to a 100 ml volumetric flask and about 70 ml of distilled water was added. The mixture was sonicated for 15 min and made up to volume by the same solvent. After filtration through a 0.45 μm syringe filter, the concentration of rizatriptan was measured according to the general procedure. The amount of rizatriptan was calculated by comparing the obtained absorbance with a standard solution at the same concentration. The same procedure was also performed for determination of rizatriptan by HPLC using a previously published method [20].

### Relative recovery

The relative recovery of rizatriptan from dosage form was evaluated by standard addition method. Rizatriptan standard solution at concentration level of 40 μg/ml was added to a solution obtained from tablet powder equal to one tablet. After ion-pair complexation and according to the general procedure, the absorbance of this solution was compared with a standard solution at 40 μg/ml after subtraction of the absorbance of a tablet powder. The relative recovery was then calculated.

### Linearity

Six series of rizatriptan solutions in the range of 0.5–50 μg/ml and also 10–100 μg/ml were prepared and the absorbance was measured according to the general procedure. The calibration curves were constructed and the statistical data were calculated. For calibration curves in the range of 0.5–50 μg/ml, 5 ml (instead of 2 ml) of rizatriptan solution were used.

### Precision and accuracy

To find out the precision and accuracy, three sets of different solutions of rizatriptan at three different concentration levels for each calibration range were prepared in triplicate and measured for 3 days. The within-day and between-day precision and accuracy were calculated.

## Results and discussion

### Absorption spectra

**Figure 2:**
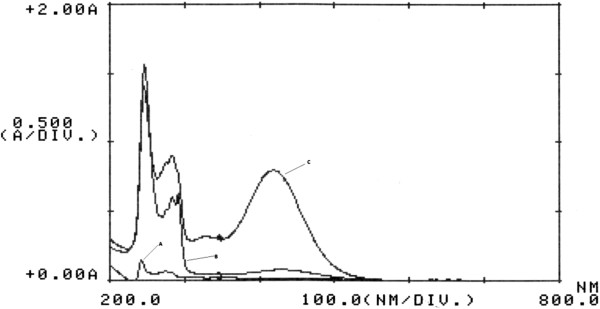
**(A) Absorption spectra of rizatriptan, (B) BCG, and (C) the ion-pair complex of rizatriptan and BCG using 2 ml of rizatriptan (5 × 10**^**-4**^ **M) and 6 ml of BCG (5 × 10**^**-4**^ **M).**

The absorption spectra of the yellow ion-pair complex between rizatriptan and BCG extracted with chloroform measured in the range of 200–800 nm against the blank solution showed a maximum absorbance at 416 nm (Figure 2). This wavelength was used for spectrophotomertic measurements. No absorption was observed in the visible region for rizatriptan or BCG solutions alone. As the maximum absorbance of the formed ion-pair complex between rizatriptan and BCG was observed in the visible region, the interference of the excipients was within the minimum range.

### Selection of the suitable pH

**Figure 3:**
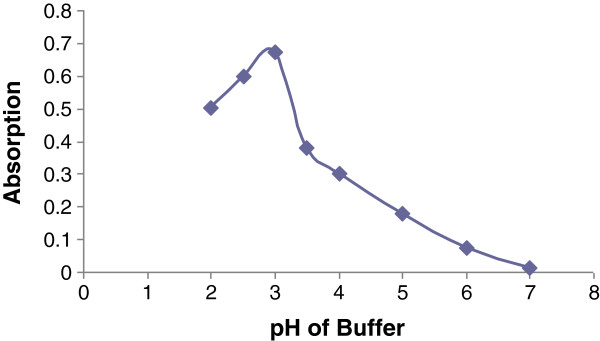
**The effect of pH of the buffer (Britton-Rabinson) on the ion-pair complex formation using 2 ml of rizatriptan (5 × 10**^**-4**^ **M) and 4 ml of BCG (5 × 10**^**-4**^ **M).**

The ion-pair complexation was performed according to the general procedure using Britton-Robinson buffer in the range of 2–7. The results are demonstrated in Figure 3. The maximum absorbance value was obtained at pH 3.0. Using different kinds of buffers (phthalate, Britton-Robinson or phosphate) at the same pH value, the best results were observed in the presence of 1 ml of phosphate buffer. It was also observed that higher absorption intensity was achieved when the buffer solution was added after rizatriptan and BCG solution.

### Selection of the reagent amount

**Figure 4:**
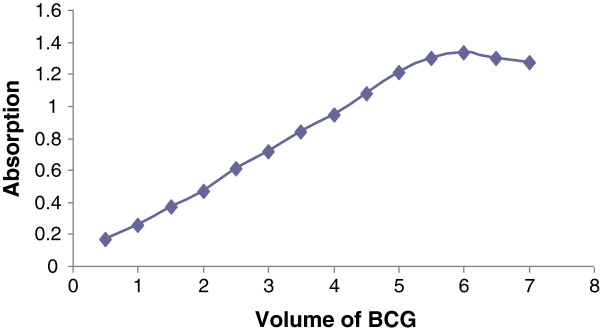
**The effect of BCG (5 × 10**^**-4**^ **M) amount on the absorbance of the ion-pair complex at 416 nm using 2 ml of rizatriptan (5 × 10**^**-4**^ **M).**

To achieve complete reaction and maximum absorbance, solutions of fixed concentrations of rizatriptan and varied volumes (0.5–7 ml) of the BCG reagent were tested. The results showed that maximum response was achieved by using 6 ml of BCG reagent (Figure 4). Higher amounts had no effect on the complex formation.

### Selection of the extracting solvent

Different extracting solvents (chloroform, dichloromethane, diethyl ether and ethyl acetate) were used. Maximum absorbance and higher selective extraction of the ion-pair complex were achieved using chloroform as the extracting solvent. It was also found that three times extraction with 5, 3 and 2 ml of solvent produced better results in comparison to one time extraction with 10 ml of chloroform.

### The effect of reaction time

Performing the ion-pair complexation in different time intervals (0, 5, 10, 20, 30 and 60 min), showed that the maximum absorbance was achieved immediately after mixing the rizatriptan solution and BCG reagent in the presence of buffer solution.

### Stability of the ion-pair complex

The stability of the rizatriptan-BCG ion-pair complex was evaluated for 24 h at room temperature. The results showed that the formed complex was relatively stable for at least 8 h (recovery > 95%).

### Composition of the ion-pair complex

The Job’s method of continuous variations by using varied volumes of equimolar solutions of rizatriptan (4 × 10^-4^ M) and BCG (4 × 10^-4^ M) was employed to find out the stoichiometry of the ion-pair complex.

**Figure 5:**
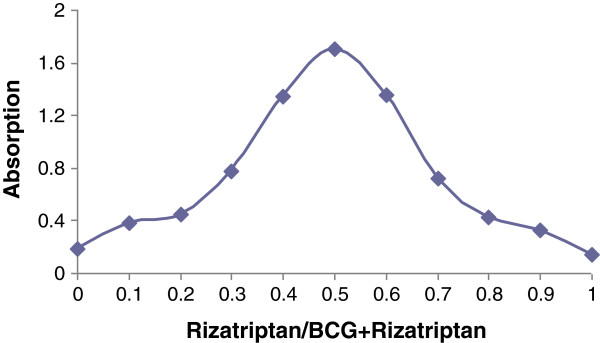
**Stoichiometry of the ion-pair complex of rizatriptan (4 × 10**^**-4**^ **M) and BCG (4 × 10**^**-4**^ **M) by Job’s continuous variation method.**

**Figure 6:**
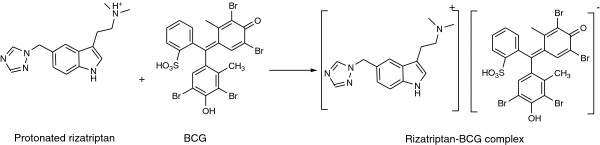
Structure of the formed ion-pair complex of rizatriptan and BCG.

The absorbance was plotted over the mole ratio of rizatriptan (Figure 5). The plot reached a maximum value at a mole fraction of 0.5 which indicated an equimolar (1:1) basis of the ion-pair complexation. The proposed structure of the ion-pair complex is displayed in Figure 6.

### Method validation

#### Linearity

The calibration curves in the range of 0.5–50 μg/ml and 10–100 μg/ml were linear according to the statistical parameters shown in Table 1. High values of correlation coefficient and small values of the intercept were achieved.

**Table 1:** Statistical data of calibration curves of rizatriptan in standard solutions (n = 6)

**Parameters**	**Low range**	**High range**
Linearity range	0.50–50 μg/ml	10–100 μg/ml
Regression equation	y = 0.025x + 0.004	y = 0.0107x + 0.0036
Standard deviation of slope	0.00033	0.00016
Relative standard deviation of slope (%)	1.32	1.53
Standard deviation of intercept	0.0015	0.0097
Correlation coefficient (r^2^)	0.9997	0.9998
Limit of Quantification (LOQ)	0.50	10.00
Limit of Detection (LOD)	0.17	3.33
Extinction coefficient (ε)	2.1 × 10^4^	2.1 × 10^4^

#### Precision and accuracy

The within-day and between-day precision and accuracy were evaluated for selected concentrations of rizatriptan in one day and three consecutive days. The results are summarized in Table 2. Low values of CV and error indicated the acceptable precision and accuracy of the proposed method.

**Table 2:** Precision and accuracy of the method for determination of rizatriptan in standard solutions (n=9; 3 sets for 3 days)

**Concentration added (μg/ml)**	**Within-day (n=3)**			**Between-day (n=9)**		
	**Found (μg/ml)**	**CV (%)**	**Error (%)**	**Found (μg/ml)**	**CV (%)**	**Error (%)**
0.50	0.49 ± 0.11	2.24	−2.00	0.49 ± 0.01	2.85	−2.00
5.00	5.01 ± 0.08	1.59	0.20	5.08 ± 0.10	1.96	1.60
50.00	50.32 ± 0.65	1.30	0.64	50.37 ± 0.88	1.75	0.74
10.00	9.86 ± 0.09	0.91	−1.40	9.94 ± 0.18	1.81	−0.60
50.00	49.83 ± 0.89	1.79	−0.34	50.33 ± 0.78	1.55	0.66
100.00	99.13 ± 1.52	1.53	−0.87	99.54 ± 1.79	1.80	−0.46

### Relative recovery

The relative recovery of rizatriptan from tablet samples was about 98 percent which showed no interferences from the excipients.

### Application of the method

The proposed method was used for determination of rizatriptan in tablet dosage forms. The proposed spectrophotometric procedure was compared with a previously published HPLC method [20]. No significant differences (p<0.05) were observed using both methods.

## Conclusion

The proposed spectrophotomertic method is relatively simple, rapid, and cost effective and it is also accurate and sensitive for determination of rizatriptan in bulk powder and tablet dosage forms. No special sample preparation is needed and no interferences from tablet excipients were observed. The limit of determination of this method is lower than other previously reported spectrophotometric methods. Therefore, the validated method could be useful for routine quality control assay of rizatriptan in pharmaceutical raw material and dosage forms.

## Competing interests

There are no other conflicts of interest related to this publication.

## Authors’ contribution

All authors contributed to the concept and design, making and analysis of data, drafting, revising and final approval. ES is responsible for the study registration. AK has done the experiments. NA provides test samples, reference material and data analysis. ES and MA are responsible for interpretation and manuscript writing and administrative support. All authors read and approved the final manuscript.
